# The impact of oral health conditions, socioeconomic status and use of specific substances on quality of life of addicted persons

**DOI:** 10.1186/s12903-015-0016-8

**Published:** 2015-03-20

**Authors:** Tais Cristina Nascimento Marques, Karin Luciana Migliato Sarracini, Karine Laura Cortellazzi, Fábio Luiz Mialhe, Marcelo de Castro Meneghim, Antonio Carlos Pereira, Glaucia Maria Bovi Ambrosano

**Affiliations:** Department of Community Dentistry, University of Campinas, Piracicaba Dental School, P.O. BOX 52, 13414-903 Piracicaba, SP Brazil

**Keywords:** Quality of life, Drug users, Substance abuse treatment, Oral health

## Abstract

**Background:**

The aim of this cross-sectional study was to evaluate the impact of oral health conditions, socioeconomic status and use of specific substances on quality of life of alcohol and drug addicted persons, receiving care at outpatient treatment facilities in Brazil.

**Methods:**

A random sample of 262 participants, mean age 37 years, from Psychosocial Care Centers for Alcohol and Drugs (CAPS AD) located in three cities in the state of São Paulo, Brazil, were clinically examined for caries experience (DMFT index) by a calibrated examiner. They were asked to complete a series of questionnaires, including the Alcohol, Smoking and Substance Involvement Screening Test (ASSIST), socioeconomic characteristics, and the World Health Organization Quality of Life assessment (WHOQOL), which were considered the outcome variables of the study. Associations between oral health status, socioeconomic characteristics, substance involvement with WHOQOL were investigated by means of the chi-square test and multiple logistic regression analysis with a level of significance α < 0.05.

**Results:**

The mean DMF index of the group was 13.0. Subjects with DMFT >14 (OR = 2.25; CI 95% = 1.30-3.89); low-income (OR = 2.41; CI 95% = 1.22-4.77) and users of cocaine/crack (OR = 2.02; CI 95% = 1.15-3.59) were more likely to have poor general quality of life.

**Conclusion:**

This study demonstrated that the general quality of life of addicted persons was associated with caries experience, low income and cocaine/crack use.

## Background

The use of illicit and licit substances has increased worldwide with important health and social consequences including the loss of people productive years and their lives [1].

According to the United Nations Office on Drug and Crime it is estimated that 243 million people aged 15–64 worldwide are users of some illicit drug, resulting in approximately 5,000 drug-related deaths per annum in Latin America and Caribbean [1].

In Brazil, data from the II the Brazilian National Alcohol and Drugs Survey indicated a prevalence of approximately 4% of marijuana use among adolescents and there are reports of increasing dependence among cannabis users [2]. With regard to cocaine and crack, 1.7% of Brazilians have used intranasal cocaine and approximately 0.8% reported smoking cocaine in the form of crack [3,4]. The prevalence of intravenous drug use in Brazil was considered very low [3,4].

Alcohol is seen by many as a more socially acceptable drug and one that has been widely used in many cultures for centuries. However, it is known that alcohol causes a large range of diseases, in addition to creating social and economic burdens on societies [4]. Various factors are associated with alcohol consumption and alcohol-related harm such as economic development, culture, availability of alcohol and the level and effectiveness of alcohol policies [4-6].

It is estimated that in Brazil there is a consumption of about 8.7 liters of pure alcohol per capita by people over the age of 15 years, and 25 percent of adults (approximately 32 million people) have some type of alcohol-related disorder [4,7]. Some national policies try to impact on alcohol consumption, such as the national legal minimum age of 18 for on-premise sale of alcoholic beverages, legally binding regulations on alcohol advertising and a national maximum legal blood alcohol concentration when driving a vehicle [4]. However, alcohol consumption continues to cause liver cirrhosis, road traffic accidents, dependence, and other social problems in Brazilian citizens [4-6].

As previously mentioned, drug and alcohol use and abuse causes great social and a financial loss to the country, including crime, domestic violence, child abuse, lost productivity, family disintegration and high risk of diseases such as HIV, Hepatitis, Tuberculosis, Cirrhosis [4-7]. Their use is influenced by gender, socioeconomic status, relationships with family and friends, culture and context [4,8,9].

Alcohol and drug addiction can have important impacts on the oral health [8-10] of these individuals. Subjects who are alcohol and drug dependent are at increased risk of having oral health compromised for various reasons, such as limited access to dental care, poor diet, poor oral hygiene habits, lack of care of oral health and general health. Furthermore, there are the effects of the substances themselves on the teeth and oral mucosa leading to bruxism, tooth loss, periodontal disease, halitosis, stomatitis and oral cancer [10-18].

Studies have shown that addiction affects the quality of life of substance users [19-25]. However, very little is known about the impact of oral health conditions on general quality of life of individuals who become addicted to alcohol and/or drugs [26].

The objective of the present study was to investigate the impact of oral health conditions, socioeconomic status and use of specific substances on the quality of life of addicted persons receiving care at?/who frequent outpatient clinics in Brazil.

## Methods

### Ethical issues

Prior to implementation, the research project was submitted to the Ethics Committee of the Piracicaba Dental School, University of Campinas, Brazil, and approved under Protocol 069/2012. Written informed consent was obtained from the participants of this study.

#### Sample population

The present cross-sectional study was conducted with a sample of adults addicted to alcohol and/or drugs, recruited from 3 multidisciplinary outpatient public clinics, located in three cities in the state of São Paulo, denominated Psychosocial Care Centers for Alcohol and Drugs (CAPS-AD), which provide services and treatment specifically to individuals with substance disorders [2].

To calculate the probability sample, we considered the situation of higher probability of sampling error (p =0.50) assuming a confidence level of 95% and a sampling error of 5%. Furthermore, we calculated the sample size for estimating the oral health of volunteers, based on data in the literature [27]. We considered an overall mean number of decayed, missing and filled teeth (DMFT index) of 14.88 with a standard deviation of 6.38 [27]. When calculating the sample, the power of test of at least 80% with significance level of 5% and minimum significant odds ratio of 1.5 in association with the variables were taken into account. There were drawn (286) 10% more than the initial sample considering possible losses.

### Clinical examination

The participants were clinically examined at the three outpatient clinics by a calibrated examiner, with reference to the presence of decayed, missing, and filled teeth in the permanent dentition (DMFT index) in an outdoor setting, under natural light with ball-point probes and mirrors, in accordance with the World Health Organization (WHO) recommendations for epidemiological surveys [28]. Before data collection began, examiner calibration was conducted by a “Gold Standard” examiner with previous experience in epidemiological surveys. Initially, a theoretical discussion was developed on guidance codes and diagnostic criteria for caries. After this, there was a practice stage including techniques for clinical examination, investigation and analysis of the results. The mean inter-examiner Kappa value of 0.95 was obtained for dental caries. The total duration of the calibration process was 28 hours. During the experimental stage, 10% of the sample of volunteers were reexamined by the same examiner in this research, to check the maintenance of diagnostic criteria and afferent intra-sampling error, and the mean kappa value of 0.89 was obtained for dental caries.

### Questionnaires

Data were collected on the participant’s sex, age, race, and socioeconomic status (monthly family income, participant’s and parents’ educational level). The questionnaires were completed in a quiet room and the interviews were realized face to face and individually.

In addition, the Alcohol, Smoking and Substance Involvement Screening Test (ASSIST) instrument [29] which was translated and validated for the Brazilian population [30,31] was applied to subjects to evaluate hazardous and harmful substance use among them over the last 3 months. The ASSIST determines a risk score for each substance, which falls into a ‘lower’, ‘moderate’ or ‘high’ risk category, and determines the most appropriate intervention for the respective level of use.

### Outcome measure

The self-administered WHOQOL-BREF is a questionnaire developed by the World Health Organization (WHO) to evaluate the general quality of life of people and it was validated in Brazil by Fleck et al. It consists of 26 questions, forming 4 subdomains about Physical Health, Psychological Health, Social Relationship and Environment Health. In addition, inside of its 26 questions, there are 2 benchmark items assessing overall quality of life and general health [32].

#### Statistical analyses

The WHOQOL-BREF scores of overall quality of life range from 0–100 and higher scores indicating better quality of life. These scores were dichotomized by the median (58.87) into lower and higher general quality of life and it represented the dependent variable being analyzed. The independent variables evaluated were sex (female and male); monthly family income (measured on the basis of the number of minimum wages –MW- the family receives, and dichotomized into “up to one MW” and “higher than one MW”), educational level of drug user and his/her parents (up to 8 years of schooling or over 8 years), race (black or white), age (dichotomized by the median into ≤ 37 years and > 37 years), median number of decayed, missed and filled teeth (dichotomized by the median into ≤ 13 years and > 13), risk of using tobacco, marijuana and cocaine/crack according ASSIST test (low risk and moderate/high risk risk).

Frequency distribution tables, crude analysis with odds ratio estimates and confidence interval of 95% were initially built to evaluate the associations between quality of life (WHOQOL) and the independent variables analyzed. Variables with p <0.20 in the crude analysis were tested in the multivariate logistic regression model, and the variables with p ≤ 0.05 remained in the model. The adjusted Odds Ratio (OR) and respective intervals of confidence of 95% were calculated for all indicators that remained in the multiple regression model at 5%. The statistical software program used to perform all analyses was SAS (SAS, 12.3).

## Results

Considering the 286 volunteers drawn initially, 24 did not agree to participate in the survey; therefore 8% was its rate of abstention, thereby arriving at a final sample of 262 subjects.

In the present study we evaluated 211 (81%) male volunteers and 51 patients (19%) were female. In Table 1 the frequency distribution of the sample is presented according to sociodemographic, economic and past caries experience variables.

**Table 1:** **Frequency of studied variables**

**Variable**	**n**	**%**
**Gender**		
Male	211	81.00%
Female	51	19.00%
**Age**		
≤ 37 years	138	52.7%
>37 years	124	47.3%
**Race**		
Black	110	42.0%
White	152	58.0%
Income*		
≤ 1 minimum wage	47	17.9%
>1 minimum wage	215	82.1%
**Subject education**		
≤ years	160	61.1%
>8 years	93	35.5%
No response	9	3.4%
**Mother’s education**		
≤8 years	208	79.4%
>8 years	29	11.1%
No response	25	95%
**Father’s education**		
≤8 years	168	64.1%
**DMFT**		
≤13	143	54.6%
>13	119	45.4%
**Alcohol use risk (ASSIST)**		
Low risk	50	19.1%
Moderate/high risk	212	80.9%
**Marijuana use risk (ASSIST)**		
Low risk	140	53.44%
Moderate/high risk	122	46.56%
**Cocaine/crack use (ASSIST)**		
Low risk	104	39.7%
Moderate/high risk	158	60.3%

*Brazilian minimum wage in effect at time of data collection = US$ 290.

Of the subjects 17.9% (n = 47) were observed to report 1 or less minimum wages as their monthly family income. With respect to education, the majority of the volunteers (61.1%), parents (64.1%) and mothers (79.4%) had up to 8 years of schooling. With respect to substance use, 80.9%, 46.56% and 60.3% of the sample were classified by ASSIST as having moderate/high risk of being alcohol, marijuana and cocaine/crack users.

Table 2 shows the results of the crude analyses and multivariate logistic regression for variables associated with general quality of life (WHOQOL). Individuals with DMFT > 13 were 2.25 times more likely to have a lower quality of life than those with DMFT ≤ 13 (CI = 1.30 to 3.89, p = 0.0039). Users classified as having high risk of using cocaine and crack by ASSIST were 2.02 more likely to have a lower quality of life compared with those at low risk (CI = 1.15 to 3.59, p = 0.0142). Among individuals receiving up to one minimum wage, 68.09% had a low quality of life, while 46.06% of volunteers earning over one minimum wage had lower quality of life. Thus, individuals living on an income of less than one minimum wage were 2.41 times more likely to present poor quality of life (95% CI = 1.22 to 4.77, p = 0.00113).

**Table 2:** **Univariate and multiple regression models for quality of life of alcohol and drug users**

	**Quality of life**									
	Low*		**High**		**Crude analysis**			**Multiple analysis**		
**Variables**	**N**	**%**	**N**	**%**	**Crude OR**	**CI 95%**	**p-value**	**Adjusted OR**	**IC95%**	**p-value**
Sex										
Male	101	38.55	110	52.13	0.64	0.34-1.19	0.160			
Female	30	58.82	21	41.18	Ref					
Age										
≤37 years	64	46.38	74	53.62	0.73	0.45-1.19	0.215			
>37 years	67	54.03	57	45.97	Ref					
Race										
Black	51	46.36	59	53.64	0.77	0.47-1.27	0.316			
White	80	52.63	72	52.63	Ref					
DMFT										
≤13	62	43.36	81	56.64	Ref			Ref		
>13	69	57.98	50	42.02	1.80	1.11-3.03	0.018	2.25	1.30-3.89	0.003
Alcohol use risk										
Low	24	48.00	26	52	0.90	0.48-1.67	0.750			
Moderate/high	107	50.47	105	49.53	Ref					
Marijuana use risk										
Low	69	49.29	71	50.71	0.94	0.57-1.52	0.800			
Moderate/high	62	50.82	60	49.18	Ref					
Cocaine/crack use										
Low	46	44.23	58	55.77	Ref			Ref		
Moderate/high	85	53.8	73	46.2	1.47	0.89-2.44	0.120	2.02	1.15-3.59	0.014
Income										
≤1 MW	32	68.09	15	31.91	2.49	1.27-4.88	0.006	2.41	1.22-4.77	0.011
>1 MW	99	46.05	116	53.95	Ref			Ref		
Subject education										
≤8 years	86	53.75	74	46.25	1.47	0.88-2.46	0.138			
>8 years	41	44.09	52	55.91	Ref					
Mother’s education										
≤8 years	105	50.48	103	49.52	1.93	0.85-4.36	0.106			
>8 years	10	34.48	19	65.52	Ref					
Father’s education										
≤8 years	84	50.00	84	50.00	1.66	0.76-3.60	0.194			
>8 years	12	37.50	20	62.5	Ref					

*Low quality of life was considered the level of reference of the dependent variable.

CI = confidence interval; Odds ratio; MW = minimum wage.

Table 3 contains a data for the mean scores of the WHOQOL-BREF and the physical, psychological, social relationships and environment domains.

**Table 3:** **Mean scores of the WHOQOL-BREF domains**

**Quality of life domain**	**Mean**	**SD**
Physical	62.08	17.70
Psychological	56.74	19.24
Social Relationship	54.77	21.09
Environment	53.75	16.69

## Discussion

Quality of life (QoL) is a multidimentional construct with interrelated domains such as physical, social, psychological and the living environment [32]. According to the World Health Organization, QoL is defined as “an individual’s perception of his/her position in life in the context of the culture and value systems in which he/she lives, and in relation to their goals, expectations, standards and concerns” [33]. Thus, QoL is influenced by sociocultural, economic, psychological and physical factors, transcending the normative vision of well-being of the biomedical model of health.

The WHOQoL-Bref has been used as generic instrument to measure quality of life without a specific correlation with any particular disease. Moreover, it comprises the satisfaction of individual with life in general, covering different domains than other health-related quality of life instruments, and it has been widely used in the area of substance use [34-36].

Drug and alcohol users have generally been found to have lower QoL than do individuals in the general population [35-37]. However, as observed in our study, the impact of addiction on QoL was also mediated by the sociodemographic conditions of users, in a trend similar to that observed in populations of non-dependent alcohol and drug users [19-37]. This is because drug users treated at CAPS-AD, who had a family income of less than or equal to one minimum wage were 2.41 times more likely to be associated with low quality of life than those from families with higher incomes. This datum was also observed in the study Moreira et al. [37], in which the authors reported that drug users, earning less than five minimum salaries, were three times more likely to have a worse quality of life compared with those with higher incomes. Therefore, the extent to which substance abuse affects QoL is affected by social determinants of health.

Despite the growing trend in studies evaluating the quality of life of drug and alcohol users [19,20,38,39], additional research investigating the associations of QOL with other classes of drugs as such marijuana are needed. In the present study, we observed that moderate and high-level users of cocaine and crack have 2.2 times more chance of presenting a worse general quality of life than low-level users and this was the only substance dependence that demonstrated significant associations with the overall scores of WHOQOL-Bref.

According to the literature, alcohol and marijuana may be less harmful to health in comparison with cocaine and crack cocaine. The latter two can cause more psychiatric comorbidities and cognitive impairment, and are also associated with sexually transmitted diseases and involvement in illegal activities, factors that can impact on health-related quality of life [1,2,40,41].

There is a scarcity of investigation into the impact of oral health on quality of life among drug and alcohol users in the literature. Wijk et al. [26] evaluated the impact of oral health on the daily functioning of a group of individuals addicted to alcohol and/or drugs, who were being treated at a specialized dentistry center in Amsterdam, and observed that the poor oral health had a substantial impact on their daily functioning. However, the authors used the short version of Oral Health Impact Profile questionnaire (OHIP-14), a specific oral health-related quality of life instrument developed to evaluate the functional and psychosocial impacts of oral diseases. Therefore, in our view, this is the first study to investigate the impact of caries experience on general quality of life by means of the WHOQOL-Brief instrument. There is an instrument to assess quality of life in drug users, but it was not validated in Brazil yet (Drug User Quality of Life Scale: DUQOL) [24]. We verified that caries experience affected the QoL of addicted persons, which led us to reflecting on the importance of the dentist as a relevant professional in the multidisciplinary teams that take care of addictive users. Dentists would help to promote oral and general health of these individuals, and contribute to their psychosocial rehabilitation, by assisting them to develop self-esteem and awareness of the possibilities of social reintegration.

The results of this study should be viewed with some limitations. It was a cross-sectional study and sought inferences with regard to causal factors without, however, establishing a temporal relationship. The self-perception questionnaire may have been influenced by social acceptance and social desirability of their addicted peers.

## Conclusion

The results of this study showed that the low quality of life of users of psychoactive substances is related to the high DMFT, low income and use of cocaine/crack. Thus, strategies with a broader approach to promoting oral health for these individuals are necessary in order to assist in the treatment of rehabilitating drug users.

## Abbreviations

ASSIST: Alcohol, Smoking and Substance Involvement Screening Test
CAPS AD: Psychosocial Care Centers for Alcohol and Drugs
DMFT: Decay, missing, filled tooth
OHIP-14: Oral Health Impact Profile questionnaire
QoL: Quality of life
SAS: Statistical Analysis Software
WHOQOL: World Health Organization Quality of Life
